# Perovskite Solar Cells Modified with Conjugated Self-Assembled Monolayers at Buried Interfaces

**DOI:** 10.3390/nano15131014

**Published:** 2025-07-01

**Authors:** Guorong Zhou, Faeze Hashemi, Changzeng Ding, Xin Luo, Lianping Zhang, Esmaeil Sheibani, Qun Luo, Askhat N. Jumabekov, Ronald Österbacka, Bo Xu, Changqi Ma

**Affiliations:** 1 i-Lab & Printable Electronics Research Center, Suzhou Institute of Nano-Tech and Nano-Bionics, Chinese Academy of Sciences, Ruoshui Road 398, Suzhou 215123, China; grzhou2023@sinano.ac.cn (G.Z.); lpzhang2012@sinano.ac.cn (L.Z.); qluo2011@sinano.ac.cn (Q.L.); 2 School of Materials Science and Engineering, Nanjing University of Science and Technology, Nanjing 210094, China; xinluo@njust.edu.cn; 3 Department of Chemistry, University of Isfahan, Isfahan 81746-73441, Iran; faezehashemi5937@gmail.com; 4 Physics and Center for Functional Materials, Faculty of Science and Technology, Åbo Akademi University, Porthaninkatu 3, 20500 Turku, Finland; ronald.osterbacka@abo.fi; 5 Department of Physics, Nazarbayev University, Kabanbay Batyr Ave. 53, Astana City 010000, Kazakhstan; askhat.jumabekov@nu.edu.kz

**Keywords:** perovskite solar cells, buried interface, self-assembled monolayer, power conversion efficiency, stability

## Abstract

In recent years, inverted perovskite solar cells (PSCs) have garnered widespread attention due to their high compatibility, excellent stability, and potential for low-temperature manufacturing. However, most of the current research has primarily focused on the surface passivation of perovskite. In contrast, the buried interface significantly influences the crystal growth quality of perovskite, but it is difficult to effectively control, leading to relatively slow research progress. To address the issue of poor interfacial contact between the hole transport-layer nickel oxide (NiOX) and the perovskite, we introduced a conjugated self-assembled monolayer (SAM), 4,4′-[(4-(3,6-dimethoxy-9H-carbazole)triphenylamine)]diphenylacetic acid (XS21), which features triphenylamine dicarboxylate groups. For comparison, we also employed the widely studied phosphonic acid-based SAM, [2-(3,6-dimethoxy-9H-carbazole-9-yl)ethyl] phosphonic acid (MeO-2PACz). A systematic investigation was carried out to evaluate the influence of these SAMs on the performance and stability of inverted PSCs. The results show that both XS21 and MeO-2PACz significantly enhanced the crystallinity of the perovskite layer, reduced defect densities, and suppressed non-radiative recombination. These improvements led to more efficient hole extraction and transport at the buried interface. Consequently, inverted PSCs incorporating XS21 and MeO-2PACz achieved impressive power-conversion efficiencies (PCEs) of 21.43% and 22.43%, respectively, along with marked enhancements in operational stability.

## 1. Introduction

Currently, high-efficiency inverted perovskite solar cells (PSCs) are primarily created through the use of SAM (self-assembled monolayer) molecules [1,2]. SAMs are highly ordered organic molecules that can spontaneously deposit onto the substrate surface through gas-phase or liquid-phase molecular assembly [3,4]. Typically, SAMs consist of an anchoring group, a linking group, and a terminal functional group. Each group plays a different role depending on its chemical composition [5]. As a result, the molecular structure of SAMs exhibits various characteristics, providing a flexible and broad design space that can be used to modify the buried interfaces of inverted PSCs [6,7].

In inverted devices, phosphonic acid- and carboxylic acid-based SAMs are commonly utilized in inverted perovskite solar cell architectures. Previous studies have shown that molecules with phosphate groups facilitate the formation of dense, uniform monolayers on various oxide surfaces through covalent bonding [8,9,10]. Based on this, Getautis et al. [11] first reported an SAM with phosphonic acid as the anchoring group, known as V1036. Inverted devices based on the V1036 SAM achieved a power-conversion efficiency (PCE) of 17.8%, indicating that using SAMs as buried interface modification layers in inverted PSCs was a promising direction. Subsequently, the development and utilization of SAMs with carbazole functional groups and phosphonic acid anchoring groups significantly improved the interface contact quality in inverted PSCs, thereby minimizing interface recombination and enhancing the PCE. More recently, Jiang et al. synthesized a novel SAM material, [4-(7H-dibenz[c,g]carbazol-7-yl)phenyl]phosphonic acid (CbzNaphPPA) [12], by replacing the flexible alkyl chain of the commonly used 4PACz with a rigid phenyl group and further extending the carbazole backbone. Compared to 4PACz, the intermolecular interactions in CbzNaphPPA are significantly enhanced, leading to the formation of H-aggregates, which substantially improve its hole mobility. Additionally, CbzNaphPPA significantly reduces defects at the perovskite buried interface and suppresses non-radiative recombination at the interface. As a result, inverted solar cells based on CbzNaphPPA exhibited an exceptionally high fill factor (FF) of 86.45% and achieved a PCE of 26.07%. Chen et al. [13] developed a mixed SAM strategy by combining the widely used [4-(3,6-dimethyl-9H-carbazol-9-yl)butyl]phosphonic acid (Me-4PACz) with an aromatic molecule, 4,4′,4″-tricyanotriphenylamine (NA), to form a mixed SAM on the surface of NiOX. On one hand, the interaction between the triphenylamine moieties in Me-4PACz and NA alleviated the aggregation of Me-4PACz, resulting in a more uniform distribution, which enhanced hole extraction and suppressed non-radiative recombination at the NiOX/perovskite interface. Ultimately, p-i-n-type PSCs employing the mixed SAM demonstrated a remarkable PCE of 26.69% and an open-circuit voltage (VOC) of 1.201 V.

Carboxylic acids are also commonly used as anchoring groups in SAMs. The presence of carboxylic acid anchoring groups helps to reduce the work function of the NiOX surface, thereby passivating the surface defects of NiOX and reducing interface energy loss [14,15]. The first two reported carboxylic acid-based molecules, TPA and MC-43 [16], were directly deposited onto ITO electrodes. These surface modifications not only enhanced hole extraction in inverted devices, but also achieved PCEs of 15.9% and 17.3%. Chang et al. [17] fabricated a functionalized SAM, poly [3-(6-carboxyhexyl)thiophene-2,5-diyl] (P3HT-COOH), to serve as a hole transport layer. They prepared the P3HT-COOH layer using spin-coating and immersion methods, and found that the immersion method was more beneficial for promoting the free rotation of the carboxyl groups in the liquid phase and their directional anchoring on the ITO surface, leading to the ordered and uniform arrangement of P3HT-COOH molecules on the ITO surface. Additionally, the prepared P3HT-COOH SAM facilitated perovskite crystallization and preferential orientation growth, which reduced defect density in the perovskite layer and enabled more efficient charge transport. The device based on the P3HT-COOH SAM achieved a PCE of 20.74%, with virtually no hysteresis. Recently, Zhou et al. [18] used a SAM molecule, TBT-BA, composed of methoxy-substituted triphenylamine-functionalized benzothiazole (TBT) and benzoic acid as the anchoring group, to modify the interface between NiOX and perovskite. TBT-BA formed a dense SAM film on NiOX, optimizing the NiOX/perovskite interface contact, promoting charge extraction, and suppressing non-radiative recombination at the interface. On the other hand, due to the relatively high binding energy of TBT-BA with perovskite, it also effectively passivated defects within the perovskite layer. As a result, inverted PSCs based on TBT-BA SAM achieved a PCE of 24.8%.

Although SAMs are widely used for modifying the buried interfaces in inverted PSCs, they still face several issues and challenges. Currently, perovskites exhibit poor wettability on most SAM substrates [19,20,21], often leading to low reproducibility and significant batch-to-batch variations [22,23]. Even on flat substrates, the uniformity of SAM films is often affected due to limited solubility of SAMs and their insufficient chemical bonding affinity with metal oxides. In this work, we developed a conjugated SAM, 4,4′-[(4-(3,6-dimethoxy-9H-carbazole)triphenylamine)]diphenylacetic acid (XS21), which contains triphenylamine dicarboxylate, to modify the interface between NiOX and perovskite. Perovskite exhibited good wettability on XS21, resulting in higher reproducibility. Utilizing these properties, we fabricated p-i-n-type perovskite solar cells with the structure ITO/NiOX/XS21/PVSK/PEAI/PCBM/BCP/Ag. The introduction of XS21 improved the crystallinity of the perovskite at the buried interface, reduced the defect state density in the perovskite, and thus suppressed non-radiative recombination. Ultimately, the XS21-based device achieved a PCE of 21.43% and significantly suppressed hysteresis in inverted PSCs.

## 2. Materials and Methods

Materials: The sodium tert-butoxide (tBuONa), tri-tert-butylphosphine tetrafluoroborate ((t-Bu)3P+BF4-), trifluoroacetic acid (TFA), and Palladium (II) acetate (Pd(OAc)2) were purchased from Sigma-Aldrich Co. Ltd. (St. Louis, MO, USA). Lead (II) iodide (PbI2) and lead (II) bromide (PbBr2), Cesium Iodide (CsI), formamidinium iodide (FAI), methylammonium bromide (MABr), phenethylammonium iodide (PEAI), and bathocuproine (BCP) were purchased from Xi’an Polymer Light Technology in China (Xi’an, China). NiOX nanoparticle powder and PC61BM were purchased from Advanced Election Technology Company in China (Shenzhen, China). MeO-2PACz was purchased from TCI AMERICA in China. XS21 was synthetized in the lab. Dimethylformamide (DMF, purity > 99%), dimethyl sulfoxide (DMSO, purity > 99%), ethanol, Isopropyl alcohol (IPA, purity > 99%), and chlorobenzene (CB, purity > 99%) were purchased from J&K Scientific (St. Jose, CA, USA). All materials were used directly.

Instruments and Characterization: The J–V characters of solar cells were measured with a Keithley 2400 source meter (Tektronix, Beaverton, OR, USA) in an N2 glove box under a simulated sun AM 1.5 G (Newport VeraSol-2 LED Class AAA Solar Simulator, Newport Corporation, Budapest, Hungary). SEM images were obtained by a field-emission scanning electron microscope (S-4800) under an accelerating voltage of 5 kV. XRD patterns were characterized using an X-ray diffractometer (Bruker-AXS D8 Advance, Bruker, Billerica, MA, USA). PL and TRPL spectra were measured using a fluorescence spectrometer (FLS1000; Edinburgh Instruments Ltd., Livingston, UK). The UV-Vis spectra were measured using a UV-Vis spectrophotometer (Lamda 750, PerkinElmer, Waltham, MA, USA).

Synthesis of di-tert-butyl 4,4′-((4-(3,6-dimethoxy-9H-carbazol-9-yl)phenyl)azanediyl)dibenzoate (2): The Buchwald–Hartwig reaction was conducted using the following materials: compound **1** (0.6 g, 1.14 mmol), 3,6-dimethoxy-9H-carbazole (0.33 g, 1.44 mmol), sodium tert-butoxide (0.24 g, 2.5 mmol), and tri-tert-butylphosphonium tetrafluoroborate (33 mg, 10% mol) were combined in a round-bottom flask equipped with a stirring bar and condenser, which included 10 mL of dry toluene. The mixture was purged and evacuated three times with argon. Next, Pd(OAc)2 (13 mg, 5% mol) was added to the mixture under an argon atmosphere. The reaction mixture was refluxed, and the progress of the reaction was monitored through TLC. After 24 h, the reaction was stopped and the toluene was removed using a rotary evaporator. The crude product was extracted with dichloromethane (3 × 25 mL). The combined organic phases were dried over MgSO_4_, followed by the evaporation of the solvent. The final product was purified by column chromatography with a hexane/ethyl acetate eluent (10:1 to 5:1) to acquire product 2 (0.54 g, 70% yield). 1 H NMR (400 MHz, CDCl3, 298 K), δ (ppm): 7.97 (d, J = 8.2 Hz, 4H), 7.59 (d, J = 2.4 Hz, 2H), 7.51 (d, J = 8.4 Hz, 2H), 7.41 (d, J = 8.4 Hz, 2H), 7.33 (d, J = 8.2 Hz, 2H), 7.20 (d, J = 8.4 Hz, 4H), 7.08 (dd, J = 8.2 Hz, J = 2.4 Hz, 2 H), 3.98 (6H, s, OMe), 1.62 (s,18H).

Synthesis of 4,4′-((4-(3,6-dimethoxy-9H-carbazol-9-yl)phenyl)azanediyl)dibenzoic acid (XS21): We poured compound **2** (0.61 g, 0.91 mmol) and trifluoroacetic acid (1.8 mL, 22.99 mmol) into a 25 mL round-bottom flask containing 10 mL of dichloromethane. The resulting mixture was stirred at room temperature for 12 h. Following this, the reaction mixture was neutralized with triethylamine. The organic phase was then extracted with dichloromethane and dried over anhydrous MgSO_4_. The pure compound XS21 was obtained with crystallization in a solvent mixture of methanol and acetone with a 64% (0.32 g) yield. 1 H NMR (400 MHz, CDCl3, 298 K), δ (ppm): 1 H NMR (400 MHz, CDCl3, 298 K), δ (ppm): 12.83 (broad, CO2H, 2H), 7.93 (d, J = 8.0 Hz, 4H), 7.84 (s, 2H), 7.62 (d, J = 7.8 Hz, 2H), 7.39 (m, 4H), 7.21 (d, J = 7.8 Hz, 4H), 7.07 (d, J = 8.0 Hz, 2 H), 3.98 (6H, s, OMe).

Preparation of device: The ITO glass substrate was cleaned by sequentially washing it with detergent, deionized water (twice), and ethanol (twice). Before use, the ITO was treated by ultraviolet ozone for 30 min. Then, a thin layer of NiOX nanoparticle film (20 mg/mL NiOX water solution) was deposited on the ITO substrate by spin-coating at 4000 rpm for 30 s, and annealed in ambient air at 150 °C for 20 min. A total of 0.5 mg/mL of MeO-2PACz and 0.7 mg/mL of XS21 was deposited on the NiOX at 4000 rpm for 30 s and annealed at 100 °C for 10 min. The Cs0.05(FA0.95MA0.05)0.95PbI3 perovskite film was prepared through dissolving 237.5 mg of FAI, 824 mg of PbI2, 25.83 mg of MAI, 13.6 mg of MACl, and 21.1 mg of CsI in mixed solvents of DMF and DMSO (*v*/*v*: 4:1). The perovskite precursor solution was spin-coated on the substrate at 5000 rpm for 30 s, 230 µL of EA was dropped onto the perovskite film at 10 s before ending the program, then the perovskite films were annealed at 120 °C for 30 min. For Cs0.05(FA0.85MA0.15)0.95Pb(I0.85Br0.15)3 perovskite films, 1.5 M of perovskite precursor solution was prepared through dissolving 190.12 mg of FAI, 548.6 mg of PbI2, 77.07 mg of PbBr2, 21.84 mg of MABr, and 17.68 mg of CsI in mixed solvents of DMF and DMSO (*v*/*v*: 4:1). The perovskite precursor solution was spin-coated on the substrate at 5000 rpm for 30 s, 230 µL of EA was dropped onto the perovskite film at 10 s before ending the program, then the perovskite films were annealed at 120 °C for 30 min. Cs0.05(FA0.98MA0.02)0.95Pb(I0.95Br0.05)3 perovskite film was prepared through dissolving 781.4 mg of PbI2, 12.9 mg of PbBr2, 277 mg of FAI, 3.8 mg of MABr, 10 mg of MACl, and 22.5 mg of CsI in mixed solvents of DMF and DMSO (*v*/*v*: 6:1). The perovskite precursor solution was spin-coated on the substrate at 5000 rpm for 30 s, 120 µL of CB was dropped onto the perovskite film at 20 s before ending the program, then the perovskite films were annealed at 120 °C for 20 min. After that, 1 mg/mL of PEAI (dissolved in IPA) was coated on the perovskite surface at 5000 rpm for 20 s and annealed at 100 °C for 10 min, then 20 mg/mL of PC61BM (dissolved in CB) was spin-coated at 3000 rpm for 30 s. Afterward, the BCP solution in IPA (0.5 mg/mL) was spin-coated on the PC61BM film at 5000 rpm for 30 s. Finally, 100 nm of the Ag electrode was thermally evaporated on the BCP film under vacuum at 5 × 10^−4^ Pa.

## 3. Results and Discussion

**Figure 1:**
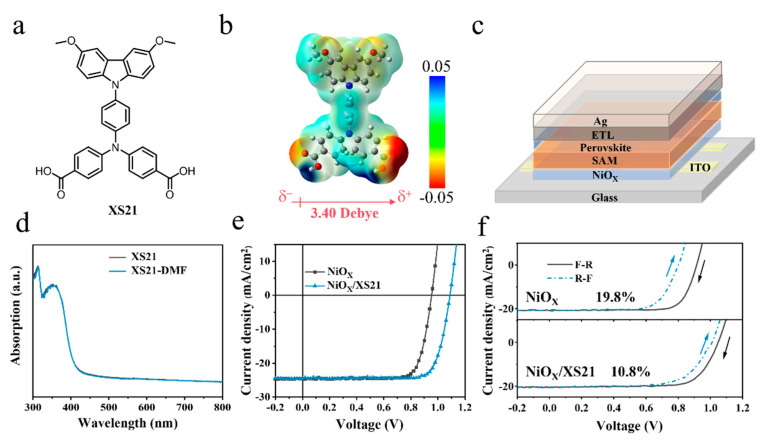
(**a**) Chemical structure of XS21. (**b**) The dipole moment of XS21. (**c**) Device configuration of inverted PSCs. (**d**) UV-Vis absorption spectra of XS21 before and after DMF rinsing. (**e**) Illuminated J-V characteristics of NiOX and NiOX/XS21-based devices. (**f**) Forward and reverse scan tests on different HTL devices.

The synthetic routes for the self-assembled monolayer XS21 are shown in Figure S1. They involved commercially available raw materials in a Pd-catalyzed Buchwald–Hartwig cross-coupling reaction with a high overall yield. The successful synthesis and purity of the molecules were confirmed by using nuclear magnetic resonance spectrometry (NMR), which are provided in the Supporting Information (Figure S2). The chemical structure of XS21 is shown in Figure 1a. The XS21 molecule contains a methoxy-substituted carbazole group, which endows the SAM with an excellent rigid conjugated plane. The connecting part of XS21 is triphenylamine, through which a stronger conjugation ability and hole transporting ability are provided for the molecule. Regarding the anchoring group, XS21 is anchored to the substrate by a carboxylic acid group. The presence of the carboxylic acid anchoring group is conducive to reducing the work function of the NiOX surface, thereby passivating the surface defects of NiOX and reducing the interfacial energy loss. Overall, the triphenylamine bridging unit and the carboxylic acid anchoring unit are introduced into the molecular structure, enabling the self-assembly of XS21 on the NiOX substrate. Subsequently, we obtained the dipole moment of XS21 using the density functional theory, which is 3.40 Debye, as shown in Figure 1b. To verify whether the SAM would be rinsed during the subsequent perovskite deposition process, we compared the UV-Vis absorption spectra of the XS21 thin film deposited on the glass substrate before and after rinsing with DMF, as shown in Figure 1d. XS21 exhibited an absorption peak in the range of 300–400 nm, and the intensity of the absorption peak remained largely unchanged after the DMF wash. This suggests that XS21 can be effectively retained during the perovskite deposition process. To investigate the modification effect of the conjugated SAM XS21 on the NiOX/perovskite interface, inverted PSCs structured with ITO/HTL/PVSK/PEAI/PCBM/BCP/Ag were fabricated, as illustrated in Figure 1c. In this study, NiOX was used as a hole transport layer (HTL) for the control group; a NiOX/XS21 mixed HTL was employed for the experimental group; and the commonly used NiOX/MeO-2PACz mixed HTL was utilized as the reference group. We compared the photovoltaic characteristics of p-i-n PSCs based on three different HTLs. Figure 1e shows the current density–voltage (J-V) curves of PSCs with the NiOX-based HTL and NiOX/XS21-based HTL. Figure S3 displays the J-V curves of PSCs with the NiOX/MeO-2PACz-based HTL. Among the photovoltaic characteristics of the three devices, the most significant difference is observed in the VOC. The highest VOC of the NiOX-based device was only 0.953 V, whereas the VOC of the MeO-2PACz and XS21-modified devices reached 1.106 V and 1.088 V, respectively. There was no significant difference in the short-circuit current density (JSC), which was 24.73 mA/cm^2^, 24.72 mA/cm^2^, and 24.51 mA/cm^2^ for the three devices. FF showed a slight improvement in the SAM-modified devices, increasing from 79.08% for the NiOX-based device to 82.07% for the MeO-2PACz-based device and 80.34% for the XS21-based device. Ultimately, when NiOX was used as the sole HTL, the highest PCE of the solar cell was only 18.63%. The NiOX/MeO-2PACz and NiOX/XS21 HTLs devices achieved PCEs of 22.43% and 21.43%, respectively. Additionally, we obtained statistical data on PCE and other J–V parameters for devices based on different HTLs. As shown in Figure S4 and Table 1, PSCs based on NiOX/MeO-2PACz exhibit a higher PCE, but the distribution ranges of their VOC, JSC, FF, and PCE values are relatively broad. In contrast, the photovoltaic parameters of PSCs based on NiOX/XS21 show narrower distribution ranges. These statistical data demonstrate the excellent reproducibility of XS21-based devices. Considering that the difference in reproducibility between the two devices might stem from the wettability of the perovskite on the SAM substrate, we conducted a contact angle analysis of perovskite precursor droplets on three different substrates. As shown in Figure S5, the contact angles for NiOX, NiOX/MeO-2PACz, and NiOX/XS21 were measured to be 31.4°, 94.3°, and 73.7°, respectively. These results indicate that the incorporation of SAMs generally deteriorates the wettability of the perovskite precursor on the substrate. However, compared to MeO-2PACz, XS21 effectively improves the wettability of the perovskite precursor, facilitating the deposition of high-quality perovskite films and consequently improving the reproducibility of devices.

Next, we performed forward and reverse scan tests on the three HTL devices to investigate the effect of different HTLs on the hysteresis of inverted PSCs, as shown in Figure 1f and Figure S6. Hysteresis presents a significant challenge in accurately assessing the PCE of PSCs. For instance, during the testing process, a significant loss of both the FF and PCE of the device was observed when scanning from forward voltage (F) to reverse voltage (R), compared to the scan from reverse to forward. It is generally believed that the hysteresis phenomenon is closely related to ion migration within the perovskite, the density of defect states, and the imbalance of charge carrier transport within the device [24,25,26,27]. The results show that the NiOX-based device exhibited the highest hysteresis. The hysteresis index (HI) is calculated by HI = (〖PCE〗_reverse-〖PCE〗_forward)/〖PCE〗_reverse, with HI reaching 19.8%. This indicates that charge carrier transport is significantly suppressed during the forward voltage scan. This could be due to the relatively low hole mobility of NiOX, which is mismatched with the electron mobility of the electron transport layer (ETL) [6,6]-phenyl C61 butyric acid methyl ester (PC61BM). On the other hand, poor interface contact between NiOX and the perovskite may result in a large number of defects. However, after modification with the two SAMs, the hysteresis phenomenon was alleviated. The hysteresis values of the NiOX/MeO-2PACz and NiOX/XS21 composite HTL devices were 8% and 10.8%, respectively.

**Figure 2:**
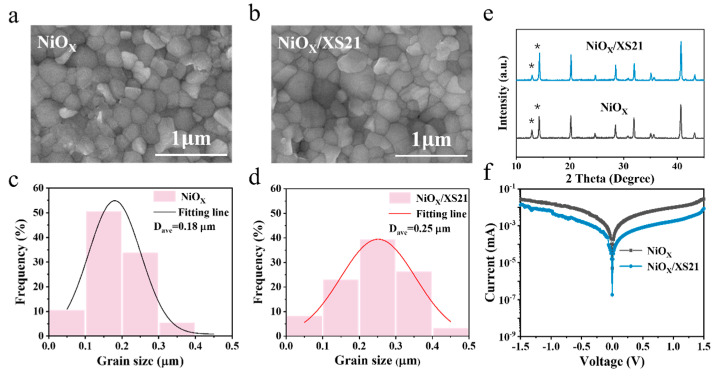
SEM images of perovskite deposited on (**a**) NiOX and (**b**) NiOX/XS21. Grain size distribution statistics of perovskite films grown on (**c**) NiOX and (**d**) NiOX/XS21 substrates. (**e**) XRD spectra of perovskite films deposited on NiOX and NiOX/XS21(The asterisks represent the diffraction peaks of PbI_2_ and FAPbI_3_). (**f**) Dark J-V curves of NiOX and NiOX/XS21-based devices.

To investigate the modification effect of SAM on the NiOX/perovskite interface, we conducted a series of characterizations on perovskite films grown on different substrates. First, we examined the morphology of perovskite films grown on NiOX and SAM substrates using scanning electron microscopy (SEM). From the SEM images in Figure 2a,b and Figure S7a, it can be seen that the perovskite crystals grown on XS21 and MeO-2PACz substrates are larger compared to those grown on NiOX. This suggests that the buried interface modification could significantly reduce the number of nucleation sites, facilitating the formation of larger grains. In addition, the grains grown on NiOX are uneven and exhibit poor flatness, whereas the perovskite films grown on SAMs are much smoother and uniform. The improved flatness further enhances the interface contact between the perovskite layer and the upper ETL, suppressing non-radiative recombination and promoting the extraction and transport of electrons to the upper layer. We further analyzed the grain size distribution of the three perovskite films and fitted the data with a Gaussian function to determine the average grain size of each perovskite film, as shown in Figure 2c,d and Figure S7b. The average grain size of the perovskite grown on NiOX is only 180 nm, while the average grain sizes of the perovskites grown on MeO-2PACz and XS21 substrates are 270 nm and 250 nm, respectively, with the largest grain size reaching 490 nm. These results indicate that after the modification of the NiOX/perovskite interface with the two SAMs, the crystallinity of the perovskite is significantly improved, resulting in perovskite films with larger and more uniform grain sizes. Subsequently, X-ray diffraction (XRD) analysis was used to characterize the crystal structure of perovskite films grown on different substrates, as shown in Figure 2e and Figure S8. Clear diffraction peaks were observed at around 13° and 14.5°, corresponding to the (001) plane of PbI2 and the (110) plane of FAPbI3. The perovskite film deposited on NiOX exhibited a higher intensity of the PbI2 diffraction peak, while the intensity of the FAPbI3 (110) diffraction peak was reduced. In contrast, after modification with MeO-2PACz and XS21, the intensity of the PbI2 diffraction peak significantly decreased, while the intensity of the FAPbI3 (110) diffraction peak was greatly enhanced. These results indicate that the SAM can passivate the interface defects at the NiOX/perovskite interface, allowing for a more complete reaction of PbI2, which in turn promotes the growth of perovskite grains and results in a better crystallographic orientation of the perovskite [28,29]. We also conducted dark J-V measurements on devices with three different HTLs, as shown in Figure 2f and Figure S9. The leakage current of the NiOX-based device was approximately 5 × 10^−6^ mA, while the leakage current of the devices modified with XS21 and MeO-2PACz was reduced by one order of magnitude. This may be attributed to the poor interface contact between NiOX and the perovskite, resulting in a less compact perovskite film and an increase in the leakage current [30,31]. However, the SAM modification significantly improves the buried interface of the perovskite, enhancing its crystallinity. This is also consistent with the SEM and XRD results.

**Figure 3:**
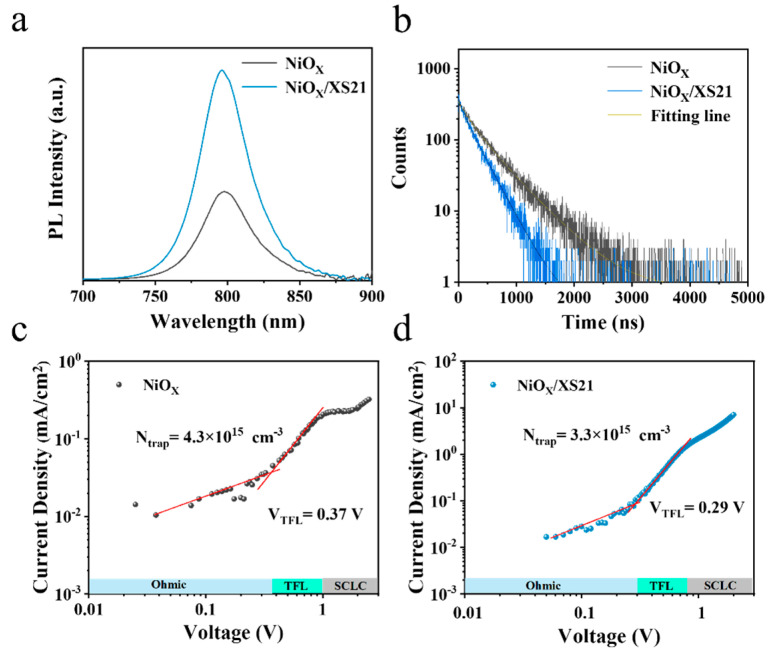
(**a**) PL spectra and (**b**) TRPL spectra of perovskite films grown on different substrates. J-V curves of single-hole devices based on (**c**) NiOX and (**d**) NiOX/XS21 HTLs.

Furthermore, Cs0.05(FA0.98MA0.02)0.95Pb(I0.95Br0.05)3 perovskite films were deposited on ITO/NiOX, ITO/NiOX/MeO-2PACz, and ITO/NiOX/XS21 substrates, followed by steady-state photoluminescence (PL) and time-resolved photoluminescence (TRPL) measurements. As shown in Figure 3a and Figure S10a, the PL emission intensity of the perovskite films deposited on NiOX is the lowest, while the PL intensity is significantly enhanced after SAM modification. The PL emission intensity of the XS21-modified film was twice that of the control group, indicating that the SAM modification of the buried interface facilitates more active exciton generation and recombination, significantly reducing non-radiative recombination. This could be due to the improved crystallinity of the perovskite after SAM modification, which reduces defect density, consistent with the SEM results. Figure 3b and Figure S10b show the TRPL spectra of perovskite films deposited on different substrates, with the corresponding fitting curves obtained using a biexponential decay formula:

(1) $$I\left(t\right)=y_{0}+A_{1}\exp\left(\frac{-t}{\tau_{1}}\right)+A_{2}\exp\left(\frac{-t}{\tau_{2}}\right)$$

In this equation, I(t) represents the PL intensity at different times; τ_1_ and τ_2_ correspond to different decay channels, where τ_1_ reflects the fast decay lifetime associated with trap-assisted charge recombination in the perovskite film; and τ_2_ represents the slow decay lifetime related to the radiative recombination process. A_1_ and A_2_ represent the weights of the corresponding decay channels. Table 2 presents the TRPL parameters of perovskite films deposited on different HTLs.

It can be seen that the fluorescence lifetime decay of the perovskite film deposited on NiOX is the slowest, while the decay accelerates after modification with MeO-2PACz and XS21. Fitting calculations reveal that the average lifetime (τavg) of the perovskite film deposited on NiOX is 447.13 ns; after modification with XS21, τavg decreases to 265.02 ns, and after modification with MeO-2PACz, τavg is 276.51 ns. The faster decay lifetimes indicate that the carrier extraction and transport at the buried interface of the perovskite are enhanced after SAM modification [32]. From Table 2, it can be observed that after SAM modification, the fraction of non-radiative recombination caused by defects A_1_ is significantly reduced, suggesting that the XS21 and MeO-2PACz substrates are more favorable for reducing the defect state density in the perovskite, thus suppressing non-radiative recombination.

Subsequently, single-hole devices with the structure ITO/HTL/Perovskite/Spiro-OMeTAD/MoO_3_/Ag were fabricated, and the effect of buried interface modification on the defect state density (Ntrap) in the perovskite was investigated using the space charge limited current (SCLC) model. By plotting the J-V curves of the single-hole devices in the dark state, the following behaviors can be observed: at a low voltage, the device exhibits Ohmic conductivity; as the voltage increases, the single-hole device enters the trap-filled limited (TFL) region; and at higher voltages, the device enters the SCLC region. The voltage transition points between the Ohmic region and the TFL region represent the trap-filled limit voltage (VTFL)(Through transition point of the fitting curves of two regions, it can be obtained), which is determined by Ntrap. Ntrap can be calculated using the following equation [33]:

(2) $$N_{trap}=\frac{2V_{TFL}\varepsilon_{0}\varepsilon_{r}}{eL^{2}}$$

where L is the thickness of the perovskite light-absorbing layer; ε_0_ is the vacuum permittivity, with a value of 8.85 × 10^−12^ F/m; ε_r_ is the relative permittivity of the perovskite, with a value of 35; and e is the elementary charge. As shown in Figure 3c,d and Figure S11, the VTFL of the NiOX-based device was found to be 0.37 V; the NiOX/MeO-2PACz and NiOX/XS21-based devices had VTFL values of 0.21 V and 0.29 V, respectively. The calculated results show that, compared to the unmodified perovskite (4.3 × 10^15^ cm^−3^), the Ntrap in the perovskite decreases significantly after SAM modification. The Ntrap of XS21-modified perovskite is 3.3 × 10^15^ cm^−3^, and for MeO-2PACz-modified perovskite, it is 2.4 × 10^15^ cm^−3^, which suppresses non-radiative recombination caused by defects [34,35].

**Figure 4:**
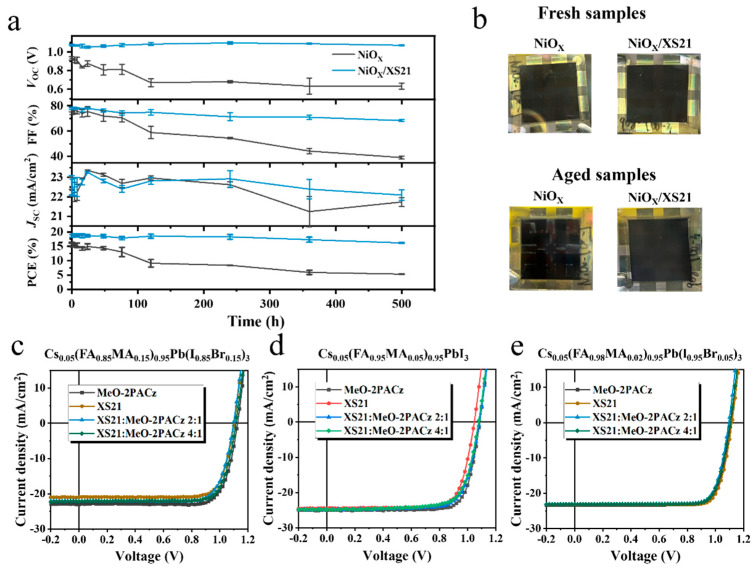
(**a**) The curves of time-dependent changes in photovoltaic parameters for devices with different HTLs during heating at 85 °C. (**b**) Photos of different HTL devices before and after thermal aging. Illuminated J-V characteristics of devices with different SAMs for (**c**) Cs0.05(FA0.85MA0.15)0.95Pb(I0.85Br0.15)3, (**d**) Cs0.05(FA0.95MA0.05)0.95PbI3, and (**e**) Cs0.05(FA0.98MA0.02)0.95Pb(I0.95Br0.05)3 perovskite compositions.

For stability, long-term thermal stability tests were conducted in a nitrogen atmosphere at 85 °C. Figure 4a and Figure S12 illustrate the changes in the photovoltaic parameters of the three different HTL devices during thermal aging. After 500 h of heating, the PCE of the NiOX-based device retained only 33% of its initial value, whereas the devices with MeO-2PACz and XS21 retained 92.4% and 87.1% of their initial PCE, respectively. Specifically, the NiOX-based device exhibited a rapid degradation of VOC and FF. In contrast, the devices modified with SAMs maintained a stable VOC during heating, with a slight improvement, while FF showed a minor decline. These results indicate that SAM modification can significantly improve the thermal stability of inverted PSCs, suppressing the rapid degradation of VOC and FF. This suggests that MeO-2PACz and XS21 can enhance the interface contact between NiOX and perovskite, mitigating interface degradation during thermal aging. Figure 4b shows photos of fresh and aged devices with different HTLs. When NiOX is used as a HTL, noticeable corrosion appears at the electrode edges after aging. In contrast, when XS21 is introduced at the buried interface, no significant change is observed at the electrode edges. This suggests that the SAM molecules can inhibit the reaction between the perovskite and the electrode, enhancing the thermal stability of inverted PSCs.

In the earlier part of this study, the application of the conjugated SAM XS21 in inverted PSCs was investigated, and it was demonstrated that XS21 could improve the interface contact between NiOX and perovskite, enhance the crystallinity of the perovskite buried interface, passivate interface defects, and subsequently improve the photovoltaic performance and stability of the inverted PSCs. However, compared to the commonly used MeO-2PACz, XS21 did not show a significant advantage. Therefore, a series of exploratory experiments were conducted to broaden the application of XS21 in inverted PSCs. Typically, the phosphate in MeO-2PACz has limited solubility and forms insufficient chemical bonds with the NiOX substrate, thus failing to achieve complete coverage on the substrate. Based on this, we proposed a mixed SAM strategy, where XS21 and MeO-2PACz were mixed to serve as the modification layer for the perovskite buried interface, to investigate its impact on the efficiency and stability of inverted PSCs. We first mixed XS21 with MeO-2PACz in different ratios to screen for the optimal mixing concentration. Table S1 summarizes the photovoltaic parameters of inverted PSCs prepared with XS21 alone as the SAM and with various mixing ratios of XS21 and MeO-2PACz. Among the four mixing ratios, a 2:1 and 4:1 mixture of XS21 and MeO-2PACz exhibited the best results, achieving PCEs of 21.36% and 21.48%, respectively. Therefore, in subsequent experiments, the 2:1 and 4:1 mixing ratios were used. Next, to verify the performance of the mixed SAM in different perovskite systems, devices were fabricated using three different perovskite compositions: Cs0.05(FA0.85MA0.15)0.95Pb(I0.85Br0.15)3, Cs0.05(FA0.95MA0.05)0.95PbI3, and Cs0.05(FA0.98MA0.02)0.95Pb(I0.95Br0.05)3. Figure 4c–e summarize the J-V curves of devices with different SAMs for each perovskite composition. The photovoltaic parameter statistics for the three compositions with different SAMs are summarized in Tables S2–S4. As shown in Figure 4c, for Cs0.05(FA0.85MA0.15)0.95Pb(I0.85Br0.15)3, the mixed SAM exhibited a slight increase in the VOC compared to the XS21-based devices, with no significant change in FF, while JSC showed a noticeable improvement. The mixed SAM devices achieved the highest PCEs of 18.99% and 19.62%, while the XS21-based device reached a maximum PCE of 18.40%. Additionally, the MeO-2PACz-based device achieved a PCE of 20.42%. In Cs0.05(FA0.95MA0.05)0.95PbI3, as shown in Figure 4d, the VOC of the XS21-based device was relatively low, and there was no significant difference in the JSC after mixing with MeO-2PACz at different ratios. However, the VOC was significantly enhanced, indicating that the mixed SAM could improve the energy level alignment between XS21 and the perovskite, reducing the hole injection barrier. Ultimately, the 2:1 and 4:1 mixed SAM devices achieved PCEs of 20.21% and 19.77%, respectively, while the XS21 and MeO-2PACz devices achieved PCEs of 19.77% and 21.30%, respectively. In the third perovskite system, Cs0.05(FA0.98MA0.02)0.95Pb(I0.95Br0.05)3, as shown in Figure 4e, the XS21-based device exhibited the highest PCE of 21.24%, surpassing both MeO-2PACz (20.98%) and the mixed SAM devices (20.44% and 20.64%), primarily due to the improvement in the VOC. This indicates that XS21 is more compatible with Cs0.05(FA0.98MA0.02)0.95Pb(I0.95Br0.05)3 perovskite, as the HOMO level of XS21 has the smallest difference with perovskite’s valence band edge.

**Figure 5:**
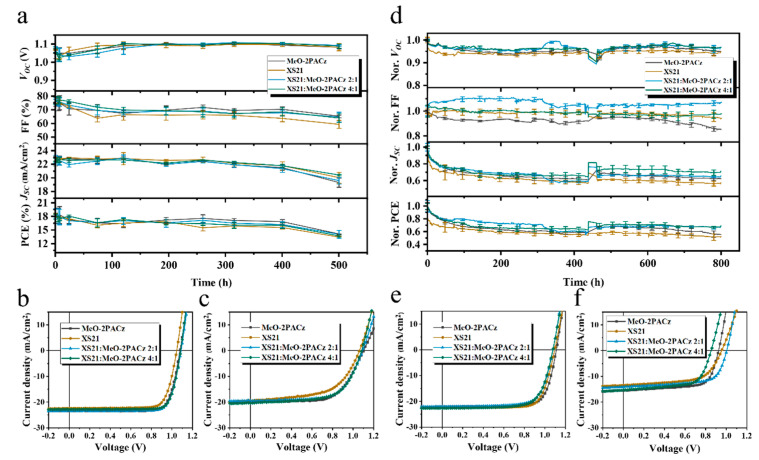
(**a**) The variation in photovoltaic parameters with time during the 85 °C heating process for the four SAM devices. (**b**) J-V curves of the fresh devices with the four different SAMs. (**c**) J-V curves of the four SAM devices after aging. (**d**) The variation in photovoltaic parameters with time during continuous operation for the four SAM devices. (**e**) J-V curves of the fresh devices with four different SAMs. (**f**) J-V curves of four SAM devices after aging.

In terms of stability, we selected the narrow-bandgap perovskite Cs0.05(FA0.98MA0.02)0.95Pb(I0.95Br0.05)3, which offers both high efficiency and stability, to compare the thermal and operational stability differences among devices with various SAM compositions. Figure 5a presents the variation in photovoltaic parameters over time during the 85 °C thermal stability test for devices with four different SAMs. The MeO-2PACz-based device retained 78% of its initial PCE after 500 h of heating, while the XS21-based device retained 72%. The devices with a 2:1 mixture of XS21 and MeO-2PACz maintained 75% of their initial PCE, and the 4:1 mixture maintained 73%. Figure 5b,c show the J-V curves before and after heating for the four SAM-based devices. It is evident that the mixed SAM does not prevent the performance degradation of the devices during the heating aging process.

Subsequently, we tested the operational stability of the four SAM devices under continuous LED illumination, with the device temperature controlled at 25 ± 5 °C. As shown in Figure 5d, the XS21-based device exhibited more significant degradation in both the VOC and JSC during the early operation compared to the other three devices, while FF remained stable. This implies that, during operation, the buried interface within the device starts to degrade, and the mobile ions within the perovskite migrate under the built-in electric field, leading to perovskite decomposition. Ultimately, the MeO-2PACz-based device retained 60% of its initial PCE after continuous operation for 800 h; the XS21-based device retained 52%; the 2:1 mixture of XS21 and MeO-2PACz retained 67%; and the 4:1 mixture retained 66%. Figure 5e,f shows the J-V curves before and after 800 h of light operation for the four SAM devices. Compared to the pure XS21 and MeO-2PACz SAMs, the mixed SAMs slightly improve the operational stability of the inverted devices.

## 4. Conclusions

In this study, a novel, conjugated SAM, XS21, was introduced to modify the buried interface of inverted PSCs. It is found that introducing XS21 at the NiOX/perovskite interface effectively passivates defects at the buried interface of the perovskite, reduces the defect state density in the perovskite, and promotes the grain growth of perovskite. This method significantly improves the interface between NiOX and perovskite in the inverted devices, leading to a remarkable enhancement in device efficiency and stability. Finally, the p-i-n PSCs structured with ITO/NiOX/XS21/PVSK/PEAI/PCBM/BCP/Ag achieved a VOC of 1.088 V and a PCE of 21.43%. In addition to using XS21 alone as the buried interface modification layer, we also attempted to mix it with MeO-2PACz to form a hybrid SAM layer. This strategy slightly improves the thermal stability of inverted PSCs, achieving a PCE of 20.64%.

In summary, the novel SAM molecule XS21 can significantly improve the interface contact between NiOX and perovskite, enhancing the overall performance of the devices, although it is still inferior to the commonly used MeO-2PACz. Based on this, we conducted a series of exploratory studies to maximize the application of XS21 in solar cells, which also provides valuable reference for the multifunctional application of SAMs in future inverted perovskite solar cells.

## Figures and Tables

**Table 1:** Statistical table of photovoltaic parameters for devices with different HTLs.

HTL	*V*_OC_ (V)	*J*_SC_ (mA/cm^2^)	FF (%)	PCE (%)
NiO_X_	0.953	24.73	79.08	18.63
	0.965 ± 0.017	24.45 ± 0.35	78.58 ± 0.70	18.52 ± 0.11
NiO_X_ /MeO-2PACz	1.106	24.72	82.07	22.43
	1.091 ± 0.016	24.66 ± 0.23	80.25 ± 3.22	21.67 ± 1.23
NiO_X_/XS21	1.088	24.51	80.34	21.43
	1.084 ± 0.014	24.44 ± 0.12	78.46 ± 1.39	20.79 ± 0.54

**Table 2:** TRPL parameters of perovskite films on different HTLs.

HTL	A_1_ (%)	τ_1_ (ns)	A_2_ (%)	τ_2_ (ns)	τ_avg_ (ns)
NiO_X_	0.39	201.61	0.61	508.35	447.13
NiO_X_/MeO-2PACz	0.29	54.89	0.71	293.43	276.51
NiO_X_/XS21	0.22	70.95	0.78	279.28	265.02

## Data Availability

The original contributions presented in the study are included in the article/Supplementary Material, further inquiries can be directed to the corresponding authors.

## Supplementary Materials

The following supporting information can be downloaded at: https://www.mdpi.com/article/10.3390/nano15131014/s1, Figure S1: Synthetic route of XS21; Figure S2: ^1^H NMR (DMSO) spectra of XS21; Figure S3: J-V characteristics of NiO_X_/MeO-2PACz-HTL device; Figure S4: Statistical results of device parameters based on 3 different HTLs; Figure S5: Contact angle analysis of the perovskite precursor droplets on NiO_X_, NiO_X_/MeO-2PACz and NiO_X_/XS21 substrates; Figure S6: Forward and reverse scan tests on NiO_X_/MeO-2PACz-HTL device; Figure S7: (a) SEM image of perovskite deposited on NiO_X_/MeO-2PACz and (b) Grain size distribution statistic of perovskite film grown on NiO_X_/MeO-2PACz substrate; Figure S8: XRD spectra of perovskite film deposited on NiO_X_/MeO-2PACz; Figure S9: Dark *J-V* curve of NiO_X_/MeO-2PACz-based device; Figure S10: (a) PL spectra and (b) TRPL spectra of perovskite films grown on NiOX/MeO-2PACz; Figure S11: *J-V* curves of single-hole devices based on NiO_X_/MeO-2PACz HTL; Figure S12: The curves of time-dependent changes in photovoltaic parameters for devices with NiO_X_/MeO-2PACz HTL during heating at 85 °C; Table S1: Statistical table of photovoltaic parameters for devices with different mixing ratios of XS21 and MeO-2PACz; Table S2: Photovoltaic parameters of devices with different SAMs in the Cs_0.05_(FA_0.85_MA_0.15_)_0.95_Pb(I_0.85_Br_0.15_)_3_ system; Table S3: Photovoltaic parameters of devices with different SAMs in the Cs_0.05_(FA_0.95_MA_0.05_)_0.95_PbI_3_ system; Table S4: Photovoltaic parameters of devices with different SAMs in the Cs_0.05_(FA_0.98_MA_0.02_)_0.95_Pb(l_0.95_Br_0.05_)_3_ system. References [36,37] are included in the Supplementary Materials.
