# Supplementary material for: Perovskite Solar Cells Modified with Conjugated Self-Assembled Monolayers at Buried Interfaces
Source: Nanomaterials (Basel). 2025 Jul 1;15(13):1014. doi: 10.3390/nano15131014 (PMC12251143; doi:10.3390/nano15131014)
Supplement: Supplementary file 1 [file nanomaterials-15-01014-s001.zip › nanomaterials-3667405-supplementary.pdf]

Supporting Information

Perovskite Solar Cells Modified with Conjugated Self-Assembled Monolayers at Buried Interfaces

Guorong Zhou [1,2], Faeze Hashemi [3], Changzeng Ding [1,4,*], Xin Luo [2], Lianping Zhang [1], Esmaeil Sheibani [3,*], Qun Luo [1], Askhat N. Jumabekov [5], Ronald Österbacka [4], Bo Xu [2,*] and Changqi Ma [1,*]

1 i-Lab & Printable Electronics Research Center, Suzhou Institute of Nano-Tech and Nano-Bionics, Chinese Academy of Sciences, Ruoshui Road 398, Suzhou 215123, China; grzhou2023@sinano.ac.cn (G.Z.); lpzhang2012@sinano.ac.cn (L.Z.); qluo2011@sinano.ac.cn (Q.L.)

2 School of Materials Science and Engineering, Nanjing University of Science and Technology,
Nanjing 210094, China; xinluo@njust.edu.cn

3 Department of Chemistry, University of Isfahan, Isfahan 81746-73441, Iran; faezehashemi5937@gmail.com

4 Physics and Center for Functional Materials, Faculty of Science and Technology, Åbo Akademi University, Porthaninkatu 3, 20500 Turku, Finland; ronald.osterbacka@abo.fi

5 Department of Physics, Nazarbayev University, Kabanbay Batyr Ave. 53, Astana City 010000, Kazakhstan; askhat.jumabekov@nu.edu.kz

* Correspondence: czding2017@sinano.ac.cn (C.D.); e.sheibani@sci.ui.ac.ir (E.S.); boxu@njust.edu.cn (B.X.);
cqma2011@sinano.ac.cn (C.M.)

Academic Editor: Peizhi Guo, Byoung-Suhk Kim, Bingbing Li and Tifeng Jiao

Received: 12 May 2025
Revised: 11 June 2025
Accepted: 16 June 2025
Published: date

Citation: Zhou, G.; Hashemi, F.; Ding, C.; Luo, X.; Zhang, L.; Sheibani, E.; Luo, Q.; Jumabekov, A.N.; Österbacka, R.; Xu, B.; Ma, C.-Q. Perovskite Solar Cells Modified with Conjugated Self-Assembled Monolayers at Buried Interfaces. *Nanomaterials* **2025**, *15*, x. https://doi.org/10.3390/xxxxx

Copyright: © 2025 by the authors. Submitted for possible open access publication under the terms and conditions of the Creative Commons Attribution (CC BY) license (https://creativecommons.org/licenses/by/4.0/).

Experimental Section

1. Synthesis processes of XS21

Chemicals: The sodium tert-butoxide (tBuONa), tri-tert-butylphosphine tetrafluoroborate ($(t\text{-Bu})_3P^+BF_4^-$), trifluoroacetic acid (TFA) and Palladium (II) acetate ($Pd(OAc)_2$) were purchased from Sigma-Aldrich Co. Ltd. The solvents used for reactions and purifications were obtained from commercial supplies that included toluene, dichloromethane (DCM), ethyl acetate, methanol, and acetone. The toluene was distilled before use; all of the other chemicals were used as received. Silica gel (100~200 mesh) was used for column chromatography. The synthesis procedure of di-tert-butyl 4,4'-((4-bromophenyl)azanediyl)dibenzoate (**1**) and 3,6-dimethoxy-9H-carbazole was similar to our previously reported literature[1,2].

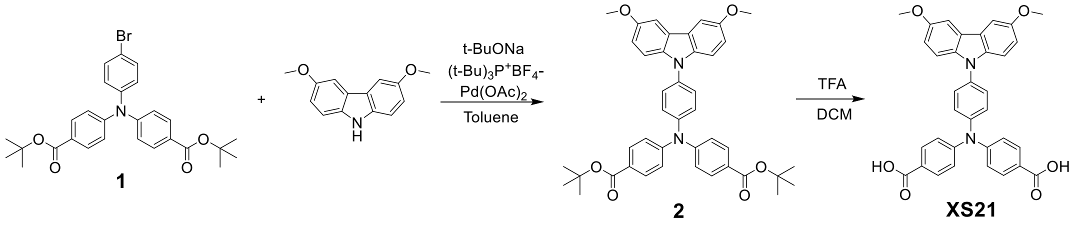

Figure S1: Synthetic route of **XS21.**

Synthesis of di-tert-butyl 4,4'-((4-(3,6-dimethoxy-9H-carbazol-9-yl)phenyl)azanediyl)dibenzoate (2): The Buchwald–Hartwig reaction was conducted using the following materials: compound **1** (0.6 g, 1.14 mmol), 3,6-dimethoxy-9H-carbazole (0.33 g, 1.44 mmol), sodium tert-butoxide (0.24 g, 2.5 mmol), and tri-tert-butylphosphonium tetrafluoroborate (33 mg, 10% mol) were combined in a round-bottom flask equipped with a stirring bar and condenser, which included 10 ml of dry toluene. The mixture was purged and evacuated three times with argon. Next, $Pd(OAc)_2$ (13 mg, 5% mol) was added to the mixture under an argon atmosphere. The reaction mixture was refluxed, and the progress of the reaction was monitored through TLC. After 24 hours, the reaction was stopped and the toluene was removed using a rotary evaporator. The crude product was extracted with dichloromethane (3 × 25 ml). The

combined organic phases were dried over $MgSO_4$, followed by the evaporation of the solvent. The final product was purified by column chromatography with a hexane/ethyl acetate eluent (10:1 to 5:1) to acquire product **2** (0.54 g, 70% yield). ^{1}H NMR (400 MHz, $CDCl_3$, 298 K), δ (ppm): 7.97 (d, J = 8.2 Hz, 4H), 7.59 (d, J = 2.4 Hz, 2H), 7.51 (d, J = 8.4 Hz, 2H), 7.41 (d, J = 8.4 Hz, 2H), 7.33 (d, J = 8.2 Hz, 2H), 7.20 (d, J = 8.4 Hz, 4H), 7.08 (dd, J = 8.2 Hz, J = 2.4 Hz, 2 H), 3.98 (6H, s, OMe), 1.62 (s,18H).

Synthesis of 4,4'-((4-(3,6-dimethoxy-9H-carbazol-9-yl)phenyl)azanediyl)dibenzoic acid (XS21): We poured compound **2** (0.61 g, 0.91 mmol) and trifluoroacetic acid (1.8 mL, 22.99 mmol) into a 25 mL round-bottom flask containing 10 ml dichloromethane. The resulting mixture was stirred at room temperature for 12 hours. Following this, the reaction mixture was neutralized with triethylamine. The organic phase was then extracted with dichloromethane and dried over anhydrous $MgSO_4$. The pure compound **XS21** was obtained with crystallization in a solvent mixture of methanol and acetone with a yield of 64% (0.32g). ^{1}H NMR (400 MHz, $CDCl_3$, 298 K), δ (ppm): ^{1}H NMR (400 MHz, $CDCl_3$, 298 K), δ (ppm): 12.83 (broad, CO_2H, 2H), 7.93 (d, J = 8.0 Hz, 4H), 7.84 (s, 2H), 7.62 (d, J = 7.8 Hz, 2H), 7.39 (m, 4H), 7.21 (d, J = 7.8 Hz, 4H), 7.07 (d, J = 8.0 Hz, 2 H), 3.98 (6H, s, OMe).

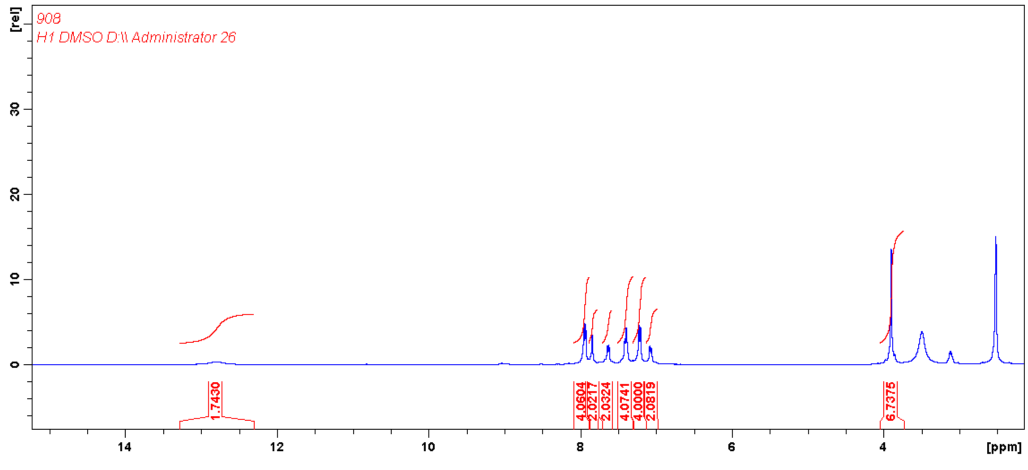

Figure S2: ^{1}H NMR (DMSO) spectra of **XS21.**

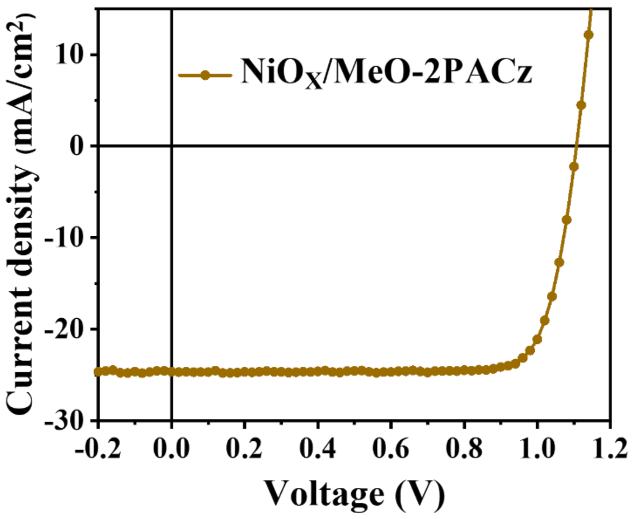

Figure S3: *J-V* characteristics of the NiOx/MeO-2PACz-HTL device.

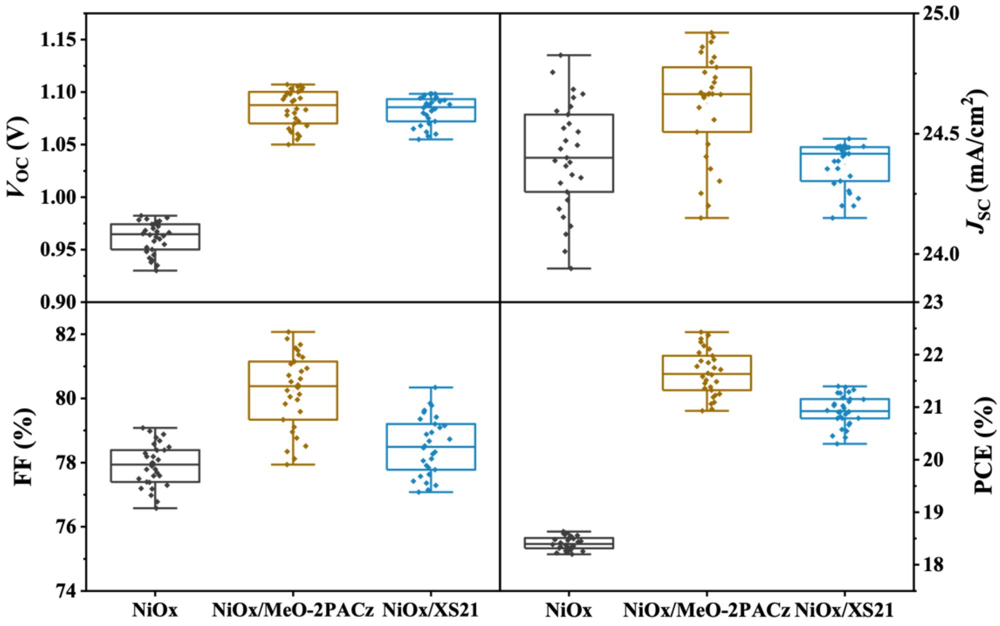

Figure S4: Statistical results of device parameters based on 3 different HTLs.

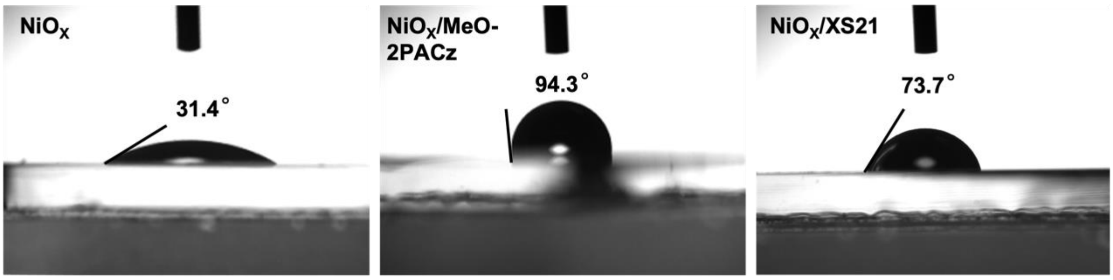

Figure S5: Contact angle analysis of the perovskite precursor droplets on NiOx, NiOx/MeO-2PACz, and NiOx/**XS21** substrates.

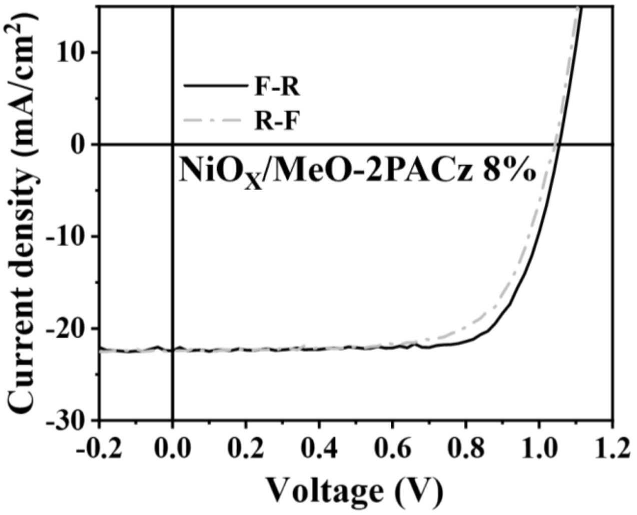

Figure S6: Forward and reverse scan tests on the NiOx/MeO-2PACz-HTL device.

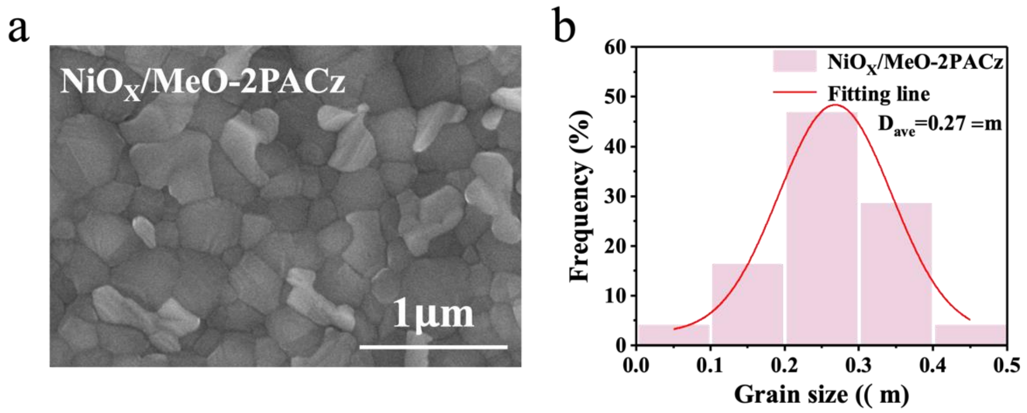

Figure S7: a) SEM image of perovskite deposit on NiOx/MeO-2PACz. b) Grain size distribution statistics of perovskite film grown on the NiOx/MeO-2PACz substrate.

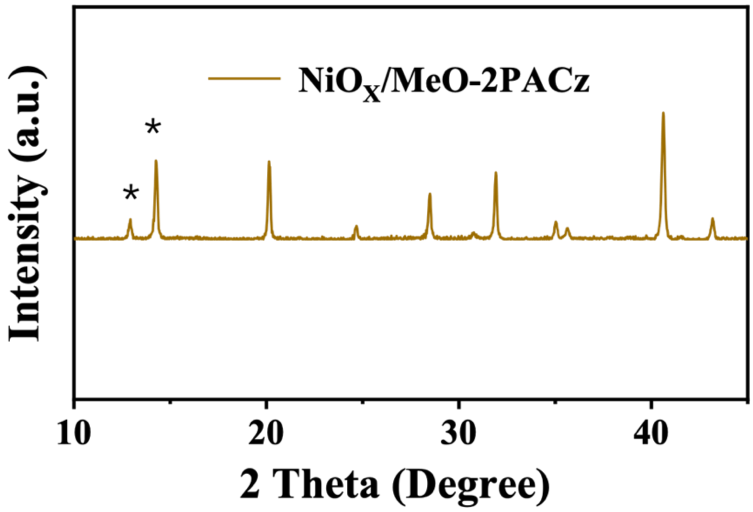

Figure S8: XRD spectra of perovskite film deposited on NiOx/MeO-2PACz.

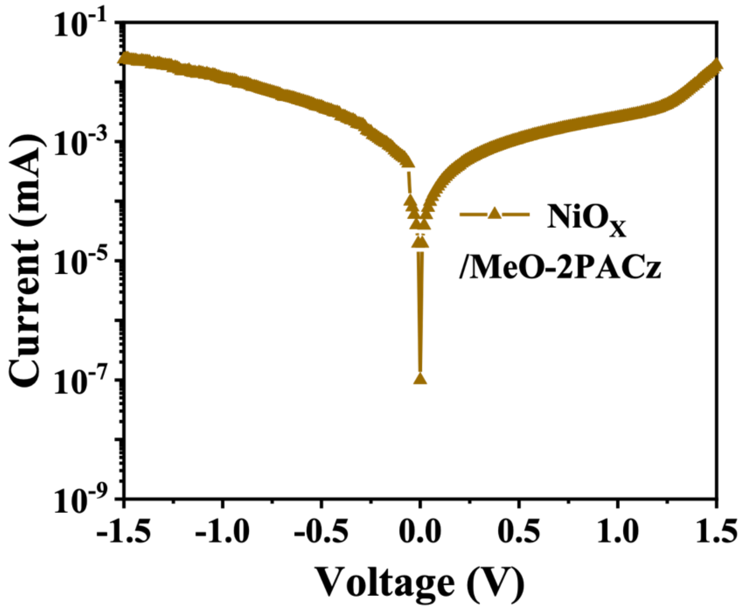

Figure S9: Dark *J-V* curve of the NiO_X/MeO-2PACz-based device.

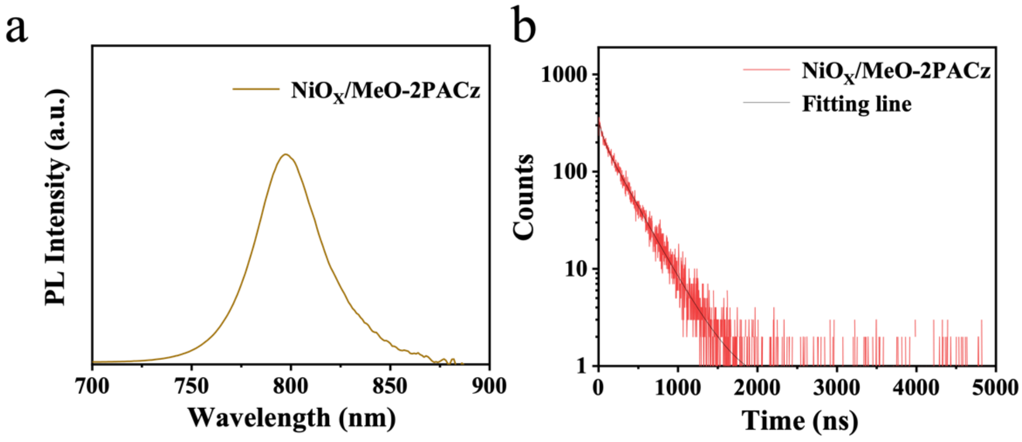

Figure S10: a) PL spectra and b) TRPL spectra of perovskite films grown on NiO_X/MeO-2PACz.

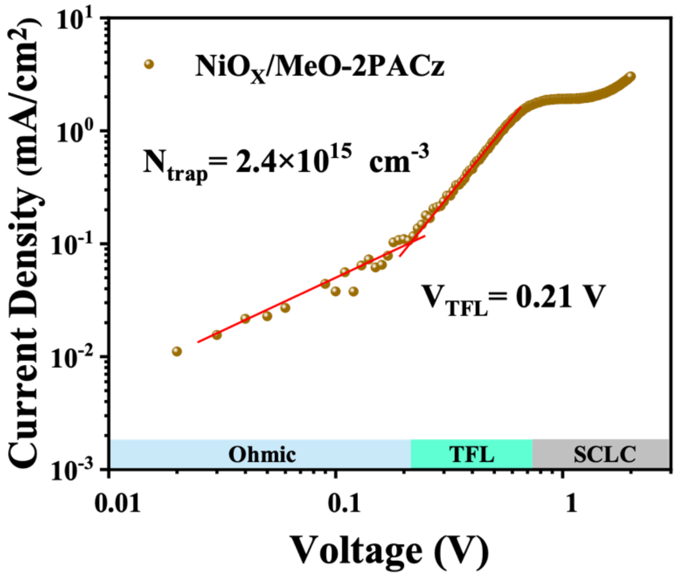

Figure S11: *J-V* curves of single-hole devices based on NiO_X/MeO-2PACz HTL.

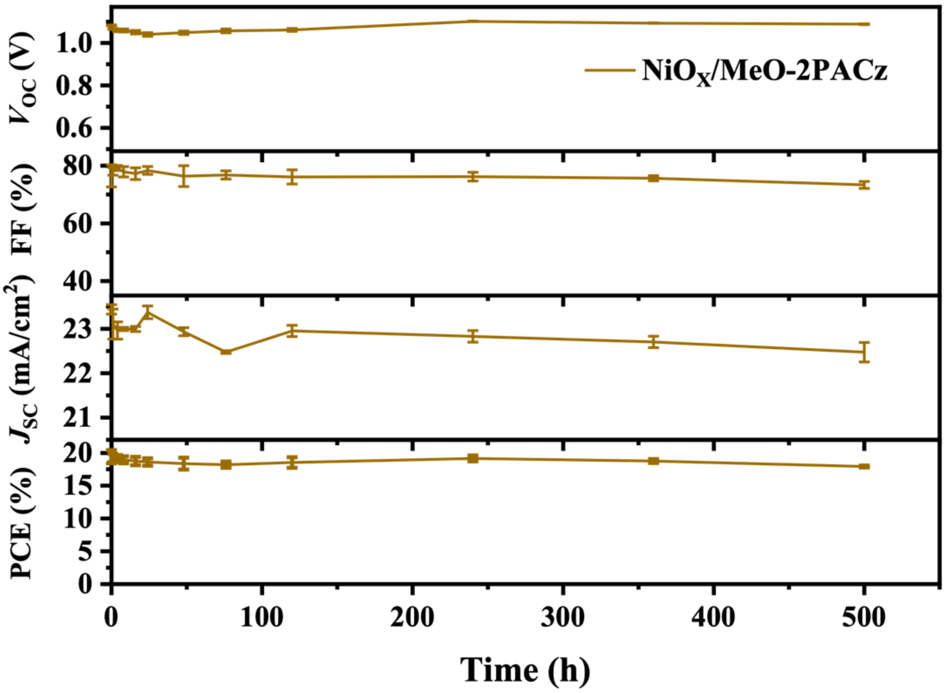

Figure S12: The curves of time-dependent changes in photovoltaic parameters for devices with NiO_X/MeO-2PACz HTL during heating at 85°C.

Table S1: Statistical table of photovoltaic parameters for devices with different mixing ratios of **XS21** and MeO-2PACz.

SAM	V_{OC} (V)	J_{SC} (mA/cm^2)	FF (%)	PCE (%)
**XS21**	1.114	24.36	80.56	21.86
	1.110±0.006	24.23±0.26	79.35±1.42	21.35±0.40
**XS21**:MeO-2PACz 1:1	1.086	24.21	77.74	20.45
	1.085±0.004	24.04±0.15	77.19±0.58	20.14±0.23
**XS21**:MeO-2PACz 2:1	1.093	24.08	79.43	21.36
	1.092±0.006	23.97±0.21	78.13±1.50	20.45±0.52
**XS21**:MeO-2PACz 3:1	1.103	24.41	79.35	20.45
	1.098±0.005	24.09±0.22	77.04±1.33	20.38±0.53
**XS21**:MeO-2PACz 4:1	1.108	23.71	81.72	21.48
	1.104±0.003	23.92±0.12	80.16±0.94	21.18±0.21

Table S2: Photovoltaic parameters of devices with different SAMs in the $Cs_{0.05}(FA_{0.85}MA_{0.15})_{0.95}Pb(I_{0.85}Br_{0.15})_3$ system.

SAM	V_{OC} (V)	J_{SC} (mA/cm^2)	FF (%)	PCE (%)
MeO-2PACz	1.125	22.91	79.28	20.42
	1.126±0.009	22.28±0.27	78.20±1.35	19.62±0.53
**XS21**	1.101	20.97	79.64	18.40
	1.099±0.011	20.49±0.72	78.62±1.12	17.70±0.61
**XS21**:MeO-2PACz 2:1	1.096	22.05	78.53	18.99
	1.106±0.009	21.19±0.72	78.76±0.47	18.46±0.40
**XS21**:MeO-2PACz 4:1	1.114	22.15	79.54	19.62
	1.105±0.016	21.75±0.57	79.25±0.70	19.05±0.42

Table S3: Photovoltaic parameters of devices with different SAMs in the $Cs_{0.05}(FA_{0.95}MA_{0.05})_{0.95}PbI_3$ system.

SAM	V_{OC} (V)	J_{SC} (mA/cm^2)	FF (%)	PCE (%)
MeO-2PACz	1.085	24.98	78.59	21.30
	1.079±0.007	24.92±0.20	76.67±2.62	20.62±0.61
**XS21**	1.046	24.37	77.58	19.77
	1.035±0.026	24.22±0.57	75.58±1.74	18.95±0.65
**XS21**:MeO-2PACz 2:1	1.082	24.75	75.44	20.21
	1.076±0.007	24.64±0.28	72.94±2.11	19.33±0.63
**XS21**:MeO-2PACz 4:1	1.077	24.79	73.88	19.71
	1.069±0.006	24.27±0.45	73.33±2.19	19.02±0.49

Table S4: Photovoltaic parameters of devices with different SAMs in the $Cs_{0.05}(FA_{0.98}MA_{0.02})_{0.95}Pb(I_{0.95}Br_{0.05})_3$ system.

SAM	V_{OC} (V)	J_{SC} (mA/cm^2)	FF (%)	PCE (%)
MeO-2PACz	1.108	23.35	81.13	20.98
	1.098±0.007	23.30±0.18	81.01±0.29	20.71±0.19
**XS21**	1.116	23.20	82.00	21.24
	1.117±0.002	22.95±0.27	81.18±0.94	20.82±0.37
**XS21**:MeO-2PACz 2:1	1.094	23.15	80.73	20.44
	1.094±0.003	22.79±0.30	80.81±0.63	20.14±0.32
**XS21**:MeO-2PACz 4:1	1.104	23.11	80.92	20.64
	1.102±0.003	22.70±0.27	80.46±0.78	20.12±0.34

Reference

1. Sheibani, E.; Zhang, L.; Liu, P.; Xu, B.; Mijangos, E.; Boschloo, G.; Hagfeldt, A.; Hammarström, L.; Kloo, L.; Tian, H. A study of oligothiophene–acceptor dyes in p-type dye-sensitized solar cells. *RSC Adv.* **2016**, *6*, 18165-18177.
2. Li, X.; Haghshenas, M.; Wang, L.; Huang, J.; Sheibani, E.; Yuan, S.; Luo, X.; Chen, X.; Wei, C.; Xiang, H. A multifunctional small-molecule hole-transporting material enables perovskite QLEDs with EQE exceeding 20%. *ACS Energy Lett.* **2023**, *8*, 1445-1454.
